# Influence of salt and temperature in the growth of pathogenic free-living amoebae

**DOI:** 10.3389/fmicb.2024.1356452

**Published:** 2024-02-15

**Authors:** Iñigo Arberas-Jiménez, Rubén L. Rodríguez-Expósito, Ines Sifaoui, Javier Chao-Pellicer, Luis Sancho, Andoni Urruticoechea, José E. Piñero, Jacob Lorenzo-Morales

**Affiliations:** ^1^Instituto Universitario de Enfermedades Tropicales y Salud Pública de Canarias (IUETSPC), Universidad de La Laguna (ULL), San Cristóbal de La Laguna, Spain; ^2^Departamento de Obstetricia y Ginecología, Pediatría, Medicina Preventiva y Salud Pública, Toxicología, Medicina Legal y Forense y Parasitología, Universidad de La Laguna, San Cristóbal de La Laguna, Spain; ^3^CEIT-Basque Research and Technology Alliance (BRTA), Manuel Lardizabal, Donostia-San Sebastían, Spain; ^4^Universidad de Navarra, Tecnun, Manuel Lardizabal, Donostia-San Sebastían, Spain; ^5^Wavegarden, Instant Sport S. L., Donostia-San Sebastían, Spain; ^6^Centro de Investigación Biomédica en Red de Enfermedades Infecciosas (CIBERINFEC), Instituto de Salud Carlos III, Madrid, Spain

**Keywords:** *Naegleria fowleri*, *Acanthamoeba*, FLA, NaCl-tolerance, temperature

## Abstract

**Introduction:**

Free-living amoebae are an extensive group of protistans that can be found in a wide variety of environments. Among them, the *Acanthamoeba* genus and *Naegleria fowleri* stand out as two of the most pathogenic amoebae and with a higher number of reported cases. *N. fowleri* is mainly found in warm freshwater water bodies whereas amoebae of the *Acanthamoeba* genus are broadly distributed through natural and anthropogenic environments. In this regard, the management and the control of the amoebic populations in swimming pools has become a major public health challenge for institutions.

**Methods:**

The aim of this work was to evaluate the growth pattern of trophozoites of *A. griffini* and *N. fowleri* at different temperatures and salt concentrations.

**Results and discussion:**

Our results showed that *A. griffini* resisted a higher concentration of salt than *N. fowleri*. Moreover, no trophozoites could withstand the salt levels of the sea in *in vitro* conditions. This work supports the contention that salinity could represent an important and useful tool for the control of the most pathogenic amoebic populations in recreational water bodies.

## 1 Introduction

Free living amoebae (FLA) are a group of protistans that are widely distributed and can be found in different environmental conditions. Some FLA can also infect humans and act as opportunistic pathogens, mostly in immunocompromised people. The amoebae of the *Acanthamoeba* genus, *Naegleria fowleri*, *Balamuthia mandrillaris*, *Sappinia diploidea* and *Vermamoeba vermiformis* have been reported as the causative agents of different human disorders such as, amoebic encephalitis, keratitis and skin and lung infection among others (Visvesvara et al., 2007; Scheid et al., 2019). The FLA infections are considered as emerging diseases since the number of reported cases is growing year by year worldwide and the incidence of climate change suggests that it is inevitable that it will continue increasing (Lorenzo-Morales et al., 2013; Maciver et al., 2020; Milanez et al., 2023). In addition, it has been described that infections caused by some species of genus *Acanthamoeba* and the specie of *N. fowleri* stand out in comparison with the other FLA infections due to the number of cases reported (Gharpure et al., 2021; Wang et al., 2023).

*N. fowleri* is found in warm and fresh water sources such as lakes, rivers or hot springs. This amoeba has not only been isolated from natural or rural areas, but is has also been found in urban environments, namely swimming pools, drinking water distribution systems or cooling waters of industries (De Jonckheere, 2012; Puzon et al., 2020). *N. fowleri* is considered a thermophilic amoeba since it grows in water sources with temperatures above 35°C and up to 46°C (Jahangeer et al., 2020). This amoeba has not been isolated from seawater, which suggests that this parasite is not able to grow at high NaCl concentrations. However, *N. fowleri* has been isolated from brackish water environment where the NaCl amount is lower (Xue et al., 2018). Previous *in vitro* studies suggest that at salinity levels above 1.5% the amoebae encyst and eventually lose their viability (Lam et al., 2019).

*N. fowleri* is the only species of its genus that is able to infect humans, causing a fulminant disease that affects the central nervous system called primary amoebic meningoencephalitis (Dereeper et al., 2022). The amoebae infect people when they swim in contaminated water bodies, entering through the nasal cavity and reaching the brain via the olfactory nerves (Grace et al., 2015).

Members of the *Acanthamoeba* genus are ubiquitous protistans that can be found in multiple environments, including water resources, thus having a direct impact with humans in everyday life (Siddiqui et al., 2016; Wang et al., 2023). These amoebae have been recognized as an opportunistic human and animal pathogen capable of causing infections in both immunocompetent and immunocompromised individuals. In this regard, *Acanthamoeba* infections most commonly manifest as a painful sight-threatening keratitis, causing an *Acanthamoeba* keratitis (AK), or a rare granulomatous amoebic encephalitis (GAE) as well as skin and lung infections (Scheid, 2016; Fanselow et al., 2021; Damhorst et al., 2022; Wang et al., 2023).

It has been reported that *Acanthamoeba* can resist many harsh environmental conditions, including temperatures from −20°C to 45°C or salinity concentrations of up to 10% (Griffin, 1979; Bergmanson et al., 2011; Solgi et al., 2012; Ramírez-Flores et al., 2023). Currently, this genus has been shown to be resistant to compounds such as fungicides and antibiotics, or chlorinating agents commonly used to disinfect swimming pools and recreational waters (Loret et al., 2008; Maycock and Jayaswal, 2016; Fanselow et al., 2021; Wolf et al., 2022). *Acanthamoeba* can also serve as hosts for endosymbionts, representing a significant reservoir for water-borne environmental pathogen/opportunistic microorganisms, such as *Legionella pneumophila* or *Aeromonas hydrophila*, and food-borne pathogens, such as *Listeria monocytogenes*, *Salmonella* spp. or *Yersinia enterocolitica*, which have important implications for human health (Loret et al., 2008; Khan and Siddiqui, 2014; Scheid, 2014; Guimaraes et al., 2016; Mungroo et al., 2021; Rayamajhee et al., 2021).

The prevalence of these FLA in different water bodies such as swimming pools or recreational water has been previously described (Chaúque et al., 2022). Therefore, there is a risk of contracting infections by these pathogenic amoebae in water. Recently, interest in the impact of salinity on recreational water safety has increased, with salinity concentrations varying from 2–3 g/L to seawater concentration (Chiswell and Wildsoet, 1989; Tiwari et al., 2021; Chaúque et al., 2022; Ramírez-Flores et al., 2023; Stahl and Olson, 2023). The aim of this study was to assess the effect of salinity concentration and temperature factors on trophozoites of these pathogenic amoebae.

## 2 Materials and methods

### 2.1 Amoebae strains and cell culture

The experiments were carried out using two amoeba species. *N. fowleri* type strain ATCC 30808™, from the American Type Culture Collection was used. The cells were axenically grown at 37°C in 2% bactocasitone medium supplemented with 10% of fetal bovine serum (FBS), 0.5 mg/mL of streptomycin sulphate and 0.3 μg/mL of penicillin G (Sigma Aldrich, Madrid, Spain). Clinical strain *Acanthamoeba griffini* genotype T3, obtained according to a previous study (González-Robles et al., 2014) was used. *A. griffini* trophozoites were grown axenically at 26°C in Peptone Yeast Glucose (PYG) medium (0.75% (w/v) proteose peptone, 0.75% (w/v) yeast extract, and 1.5% (w/v) glucose) containing 40 μg of gentamicin mL^−1^ (Biowest, Nuaillé, France).

### 2.2 Amoebae growth *in vitro* assays in saline bactocasitone/PYG mediums

For the growth assays in saline bactocasitone (*N. fowleri*) and PYG medium (*A. griffini*), four different salt concentrations were used: 1, 5, 15 and 34 g/L (sea water salt concentration) (Chubarenko et al., 2023; U.S. Geological Survey, U.S. Department of the Interior, 2024). The experiment started with the media change from fresh bactocasitone/PYG medium to the same growth media with the different NaCl concentrations and incubation of the trophozoites (1 mL) in a 24 well plate at a starting concentration of 10 cells/mL for *N. fowleri* and 100 cells/mL for *A. griffini*. The incubation was also done at three different temperatures: 20°C, 28°C and 37°C. In addition, *N. fowleri* cells were also incubated at 26°C. The number of cells that were present in each well was measured at 24, 30, 48, 54 and 72 h post incubation using the EVOS M5000 Cell Imaging System (Life Technologies, Madrid, Spain). The experiments were performed in triplicate and the results are expressed as the mean value of both assays.

### 2.3 Amoebae growth *in vitro* assays in saline tap water

The growth assays in saline tap water were performed following a very similar protocol to the one mentioned above. Firstly, the tap water was filtered with a 0.45 μm filter. After, the marine salt was diluted at 1, 5, 15 and 34 g/L. The growth media of the trophozoites was changed in order to add the different saline water dilutions. Finally, a concentration of 100 cells/mL was seeded (1 mL) in a 24 well plate. The same temperatures as in the previous experiments were used. The number of cells were determined at 24, 30, 48, 54 and 72 h after the incubation of the trophozoites in the EVOS M5000 Cell Imaging System (Life Technologies, Madrid, Spain). The experiments were performed in triplicate and the results are expressed as the mean value of each assay.

### 2.4 Statistical analysis

Statistical comparison using two-way analysis of variance (ANOVA) was conducted to highlight the differences in amoebic population growth between the tested experimental conditions of temperature, salinity concentration and time for *Acanthamoeba* and *Naegleria fowleri, p* < 0.05 was considered statistically significant. Statistical analyses and the graphs were carried out using GraphPad Prism 9.0. program (GraphPad Software, San Diego, CA, United States). All the data were attached to Supplementary material.

## 3 Results

### 3.1 Evaluation of the effect of different salt concentrations against *N. fowleri* and *A. griffini*

**Figure 1:**
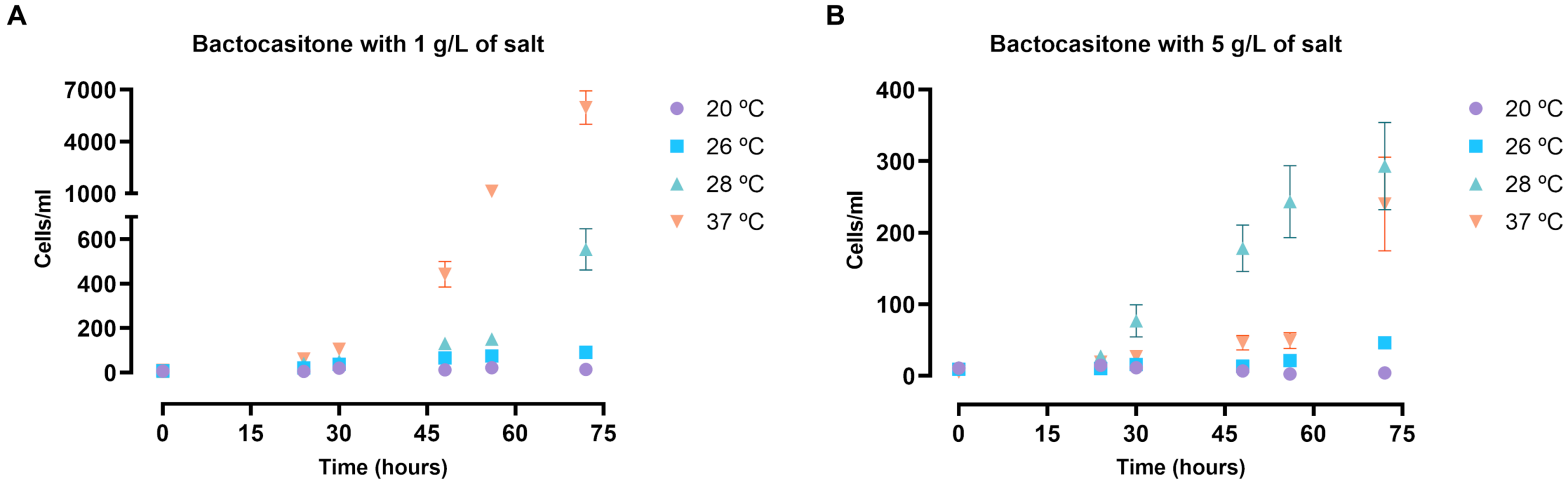
*N. fowleri* trophozoite growth at 1 **(A)** and 5 **(B)** g/L of salt diluted in bactocasitone medium incubated at four different temperatures (20, 26, 28, 37°C). The cell counting was performed at 24, 30, 48, 56 and 72 h, starting at a concentration of 10 cells/mL. Data of the experiments at 15 and 34 g/L of salt is not shown since no viable trophozoites could be observed after 24 h. Each data point represents the mean ± standard deviation (SD) of three different measurements. In both cases, very little cell growth was observed when incubating them at 20 and 26°C. The number of trophozoites increased exponentially at 1 g/L of salt and 37°C, while at 28°C the amoebic concentration also augmented, but at a slower rate. At 5 g/L the cell growth was similar at both 28 and 37°C.

A first approach of the viability of *N. fowleri* trophozoites in salt medium was performed in bactocasitone. Figure 1 shows the results of the amoebic growth at four salt concentrations (1 g/L, 5 g/L, 15 g/L, 34 g/L) and incubation at different temperatures (20°C, 26°C, 28°C and 37°C). After 24 h, no viable amoebae could be observed at 15 g/L nor 34 g/L. The cellular growth was slower at higher salt concentrations. The temperature had also an important influence on the trophozoite development. There was barely any cell growth in low temperature (20 and 26°C) incubated amoebae. At the 1 g/L salt concentration the increase in the number of trophozoites was remarkably higher at 37°C (*p* < 0.0001, comparing the number of cells at 0 and 72 h, Supplementary Table S1A) than at 28°C (*p* < 0.05) (Figure 1A), while at 5 g/L the amoebae concentration at 72 h of incubation was similar in both temperatures. Beside this, at 37°C a significant increase in cells number was observed between 56 and 72 h (*p* < 0.0001, Supplementary Table S1A) (Figure 1B).

**Figure 2:**
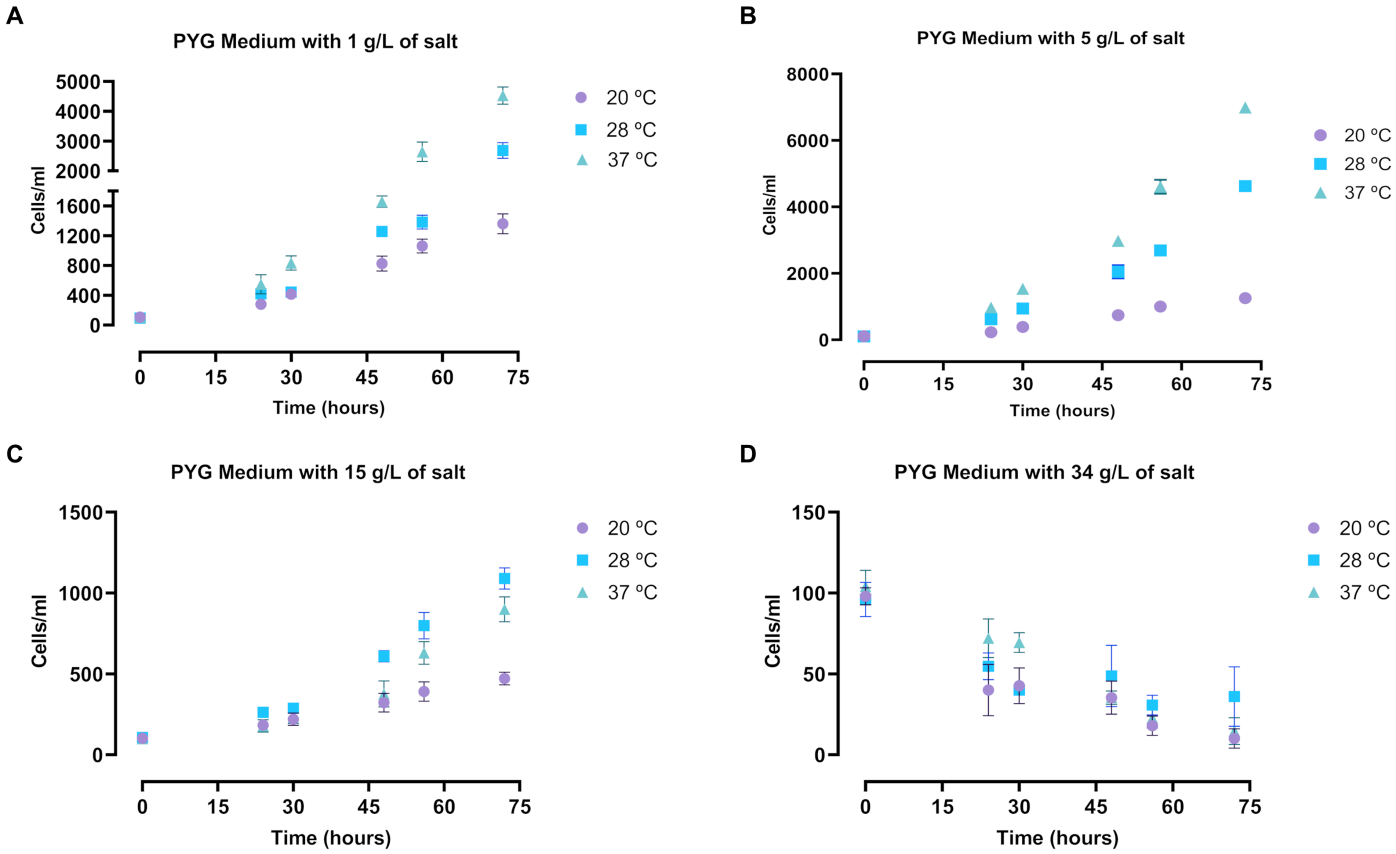
Determination of the growth of *Acanthamoeba griffini* trophozoites cultured in PYG medium with four different salt concentrations: 1 g/L **(A)**, 5 g/L **(B)**, 15 g/L **(C)** and 34 g/L **(D)**, at 20, 28 and 37°C for 72 h, starting at a concentration of 100 cells/mL. Each data point represents the mean ± standard deviation (SD) of three different measurements. An increase of the cell population was observable at 1, 5 and 15 g/L after 72 h, while at 34 g/L the number of cells decreased.

Likewise, a very similar assay was performed in trophozoites of *A. griffini,* incubated in salty PYG medium. In general, the *Acanthamoeba* trophozoite population grew in the different salt concentrations and temperatures after 72 h (Figure 2). Culture in a salt concentration of 34 g/L significantly decreased the amoebae population in all three temperatures (*p* < 0.0001, Supplementary Table S2A) (Figure 2D), suggesting that at this salt levels trophozoites of *A. griffini* cannot resist the osmotic pressure and started to encyst after 72 h of incubation. For incubations with 1 and 5 g/L of salt (Figures 2A,B) we observed that trophozoite population showed a significant increase comparing with 15 and 34 g/L salt levels (*p* < 0.0001, Supplementary Table S2A) (Figures 2C,D). In addition, there were statistically significant differences between the three temperatures. Trophozoites incubated at 28 and 37°C exhibited an increase in amoebae population compared to cells incubated at 20°C after 72 h (*p* < 0.0001, Supplementary Table S2A) (Figures 2A–C), highlighting amoebae growth at 37°C in the experiments with 1 and 5 g/L of salt levels.

**Figure 3:**
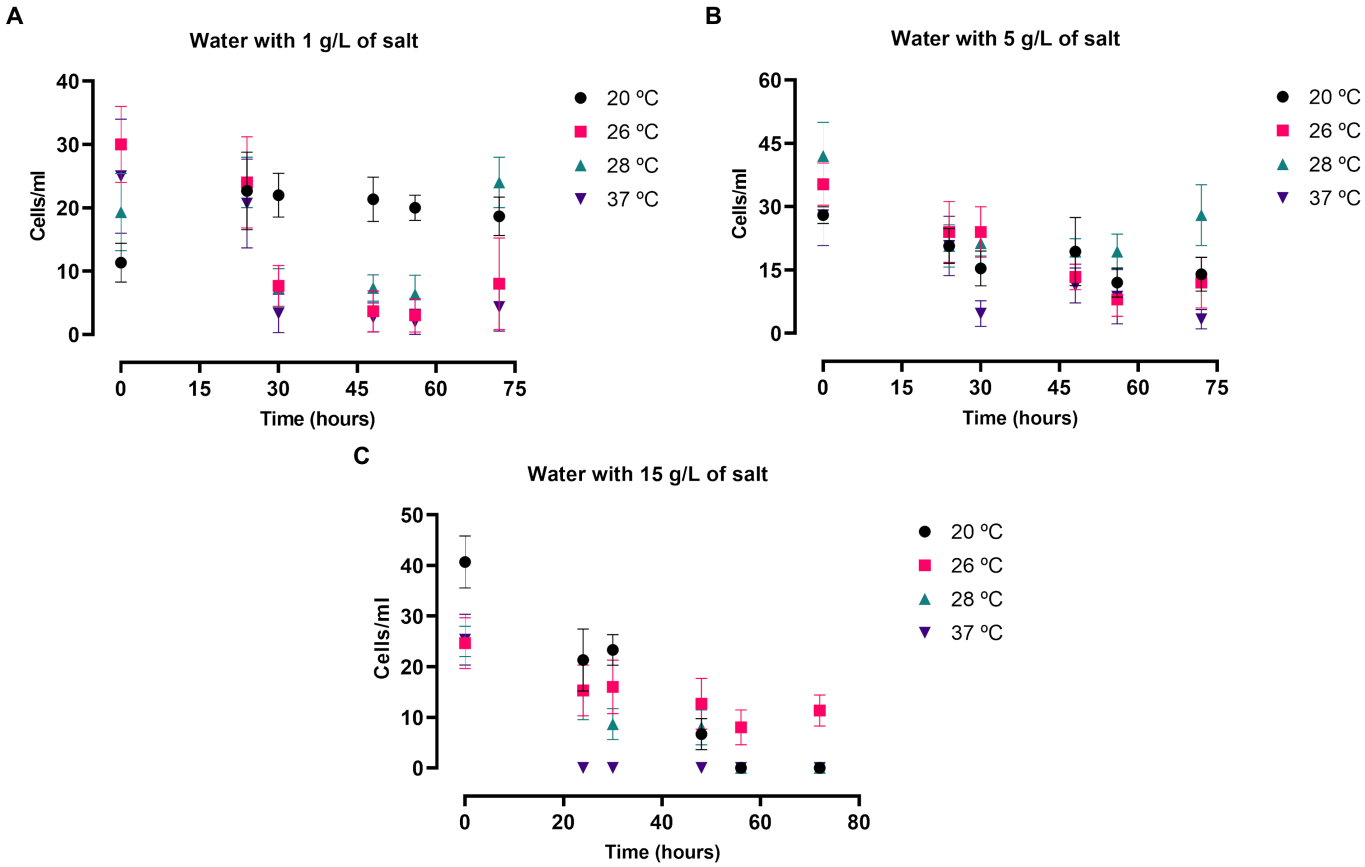
*Naegleria fowleri* trophozoite growth in saline water at 1 **(A)**, 5 **(B)** and 15 **(C)** g/L of salt incubated at 20, 26, 28 and 37°C. The number of cells was measured at 0, 24, 30, 48, 54 and 72 h. Each data point represents the average cell population of three different experiments ± SD. At 28°C the cellular concentration remained similar after 72 h of incubation at 1 and 5 g/L. Moreover, the number of cells persisted stable all along the experiments at 20°C and 1 g/L. Overall, the number of cells decreased, reaching to 0 viable cells in some cases, in the rest of the tested conditions.

The amoebic viability and growth in culture was also evaluated in salty water in both FLA species. The assay was performed at the same salt concentrations and incubation temperatures as mentioned in the previous experiments, but in these experiments cells were cultured in water medium without nutrients. The results in *N. fowleri* are summarized in the Figure 3 and Supplementary Table S1B. The number of cells that could be observed in these experiments was remarkably lower than when incubated in bactocasitone after 72 h (*p* < 0.0001, Supplementary Table S1C), except for cells incubated at 20°C and 1 g/L of salt. At this temperature no amoebic growth was observed in both nutrient-rich and nutrient-poor assays. In salty water, amoebae did not grow after 72 h of incubation in any case. Only at 20 and 28°C the amount of trophozoites at 0 and after 72 h remained similar at 1 g/L of salt concentration. At the highest salt concentration (34 g/L) no viable cells were observed after 24 h. At 15 g/L, only at 26°C some live cells could be observed after 72 h (Figure 3C), while no viable cells were detected at the rest of the incubation temperatures. At the lowest salt concentrations 1 and 5 g/L, the amoebic population significantly decreased in most of the tested conditions after 72 h of incubation (*p* < 0.05 and *p* < 0.0001, Supplementary Table S1B) (Figures 3A,B). Moreover, at 1 g/L at 20 and 28°C the number of cells remained stable throughout the experiment.

**Figure 4:**
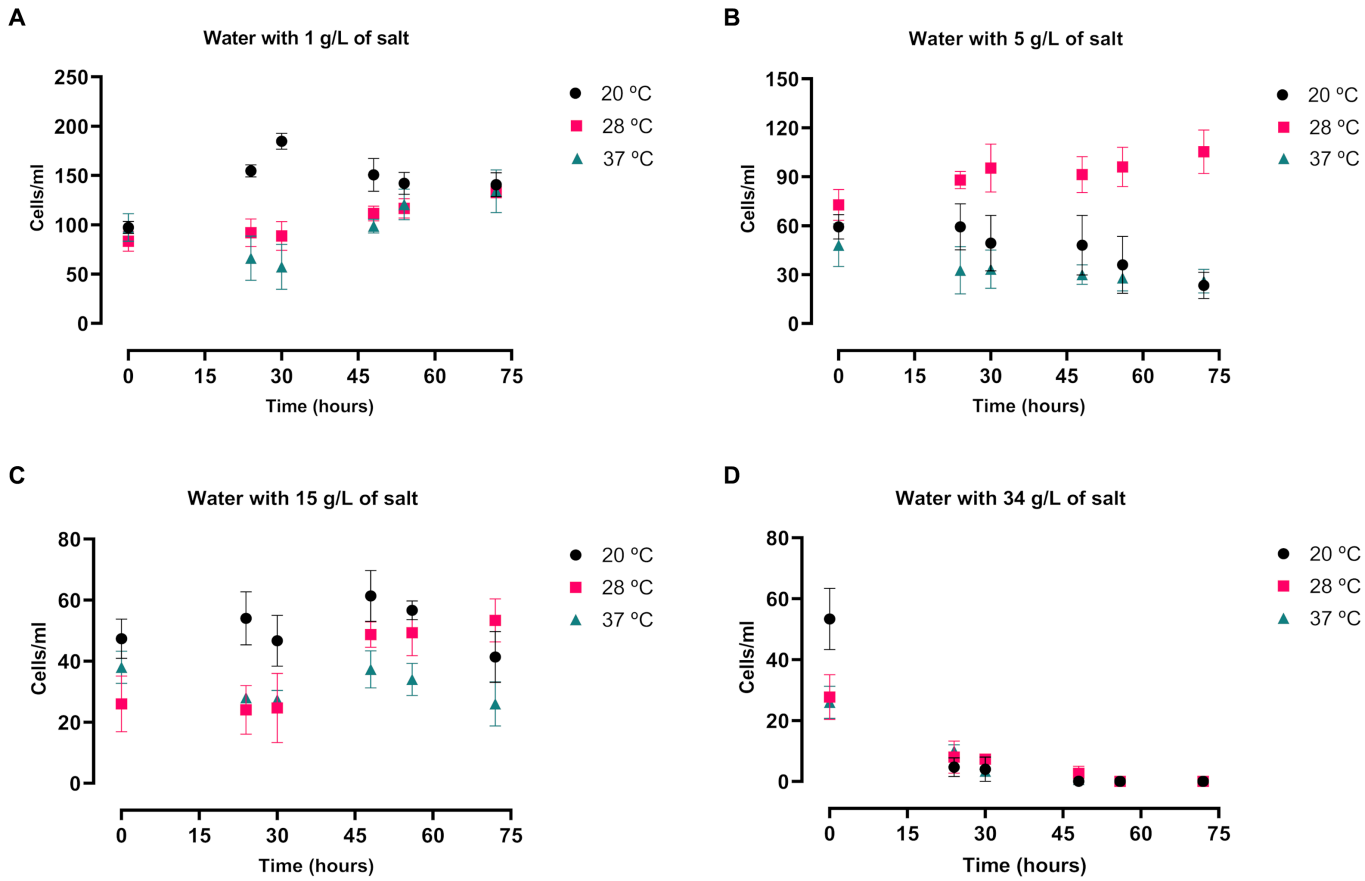
Evaluation of the *Acanthamoeba griffini* trophozoites growth cultured in saline water with four different salt concentrations: 1 g/L **(A)**, 5 g/L **(B)**, 15 g/L **(C)** and 34 g/L **(D)**, at 20, 28 and 37°C for 72 h, starting at a concentration of 100 cells/mL. Each data point represents the mean ± standard deviation (SD) of three different measurements. A conservative growth was observed in the salt concentrations of 1, 5 and 15 g/L, whereas at the higher salt level no amoebic population was detected after 48 h.

*A. griffini* trophozoites cultured in filtered tap water in different salt concentrations and temperatures showed a conservative growth (Figure 4). In comparison with the other salt concentrations, cells incubated with 1 g/L of salt level presented a higher number of trophozoites after 72 h (*p* < 0.0001 and *p* < 0.001, Supplementary Table S2B), obtaining similar results in all three temperatures (Figure 4A). Beside this, amoebae cultured with 5 and 15 g/L of salt concentration presented similar number of amoebae after 72 h (Figures 4B,C), highlighting the cells incubated at 28°C with 5 g/L of salt level that demonstrated a significantly higher number of cells in comparison with 20 and 37°C (*p* < 0.0001, Supplementary Table S2B). Nonetheless, trophozoites cultured with 34 g/L of salt concentration did not show any growth after 48 h of incubation (*p* < 0.0001, Supplementary Table S2B) (Figure 4D), possibly due to that at this higher concentration of salt in tap water incubation the osmolarity pressure is much higher. Hence, trophozoites of *A. griffini* were unable to grow. Nor were they able to grow in PYG medium supplemented with 34 g/L of NaCl. In addition, amoebae incubated in PYG medium showed a significantly higher number of cells in comparison with trophozoites cultured in salty water after 72 h (*p* < 0.0001, Supplementary Table S2C).

## 4 Discussion

Free living amoebae are well known for being ubiquitous organisms and hence being able to survive in different environmental conditions. Their presence in different water bodies has become an important issue to deal with for public authorities, becoming even more concerning when considering recreational water facilities where the parasites come into contact with the bathers.

The aim of this study was to evaluate the *in vitro* effect of different salt concentrations and temperatures in the growth of small populations of the pathogenic FLA of the *Acanthamoeba griffini* and *Naegleria fowleri* species. The NaCl has already proved to have an impact on different bacterial communities altering their growth patterns (Pang et al., 2020; Li et al., 2021). Interestingly, it has also been proposed as an alternative treatment for the human cutaneous leishmaniasis caused by the protozoa *L. major* (Abbas Marhoon and Majid, 2023). In this context, the use of NaCl to control the amoebic population in recreational water facilities could represent a complementary option to the currently available disinfection agents. *N. fowleri* grows preferentially in warm water, above 25°C, and some countries consider water supplies above this temperature to be dangerous because of the possible presence of this parasite (Cabanes et al., 2001; Water Services Association of Australia, Water Research Australia, 2016). Hence, we considered it appropriate to add a fourth experimental temperature (26°C) in the case of *N. fowleri*. To date, the available data regarding the amoebae growth at different experimental conditions has been carried out at high amoebic concentrations (Griffin, 1979; Bergmanson et al., 2011; Lam et al., 2019; Stahl and Olson, 2023). However, the number of cells that are usually found in swimming pools is far from those tested concentrations (Chaúque et al., 2022). For instance, French authorities only allow a maximum of 100 *N. fowleri* cells per liter (Cabanes et al., 2001; Agence nationale de sécurite sanitaire alimentation, enviroment T, 2013).

Results using *N. fowleri* show that the salt concentration has a negative effect on its growth since the number of viable cells decreases as the salt amount increases for both nutrient-rich and nutrient-poor experiments. As expected, due to the nutrient composition of the medium, in the bactocasitone and salt cultured cells the growth was much higher compared to the water and salt incubated cells. For this reason, we decided to start the experiments in bactocasitone at a 10-fold lower concentration. Furthermore, there were notable differences when culturing the cells at higher temperatures, where the growth was larger (*p* < 0.0001, comparing the number of cells at 0 h and 72 h, Supplementary Table S1A), than at lower temperatures, where the cell population was barely the same after 72 h as at the beginning of the assay. Beside this, no viable cells could be observed after 24 h of incubation of bactocasitone with 15 g/L and 34 g/L of salt, while at 15 g/L of salt diluted in water some trophozoites still remained viable. This could be explained by the low number of cells that were incubated in the bactocasitone assay (10 cells/mL). As it can be seen in the experiment performed at 15 g/L of salt in water the cell population halved after 24 h, however the starting number of cells was 100 cells/mL and trophozoites could be measured. These results are in line with previous work where the tolerance of *N. fowleri* up to 5 g/L of salt is described, finding less viable cells as the salt concentrations increases. In addition, in all the studies salt concentrations above 15 g/L inhibited the growth of *N. fowleri* trophozoites (Lam et al., 2019; Stahl and Olson, 2023).

Trophozoites of *A. griffini* cultured in different NaCl concentrations and temperatures showed similar results as *N. fowleri.* At 34 g/L of salt concentration almost no viable cells were found for both assays incubated with PYG medium and tap water after 72 h in *A. griffini*. Only at 28°C with PYG medium some cells could be observable. In addition, at the 34 g/L NaCl level the temperatures did not influence the growth of the trophozoites. Therefore, these results suggest that at this salt concentrations trophozoites cannot resist the osmotic pressure, consequently some amebic population showed an early stages of encystation process. In this regard, Ramírez-Flores et al. (2023) isolated different strains of *Acanthamoeba* from samples of a geothermal power plant to assess the salinity tolerance of these genera pathogenic amoebae (Ramírez-Flores et al., 2023). They showed that isolated amoebae presented a great tolerance to high concentrations of salts, including levels of 5.56% of salinity and temperatures up to 42°C. Beside this, Bergmanson et al. (2011) evaluated the *in vitro* effect of different NaCl levels with PYG medium and distilled water against trophozoites of *A. castellanii* and *A. polyphaga* (Bergmanson et al., 2011). In that study, authors showed that amoebae cultured with a 10% of NaCl concentration were between 83 and 100% in cyst stage after 19 days of incubation for both tested strains. All these results confirm the high resistance to extreme conditions of some strains of amoebae belonging to the *Acanthamoeba* genus.

In contrast, *A. griffini* trophozoites incubated at salt levels of 1, 5 and 15 g/L showed differences when culturing them in PYG medium or tap water. Major number of cells were observed in all salt concentration of PYG medium incubations in comparison to tap water experiments after 72 h (*p* < 0.0001, Supplementary Table S2C), possibly due to the higher presence of nutrients in the PYG medium assays. Furthermore, the highest growth of trophozoites cultured with temperatures close to 28 and 37°C in both nutrient-rich and nutrient-poor experiments, evidenced that *Acanthamoeba* prefers warm environments for growth (Scheid, 2016; Fanselow et al., 2021; Wang et al., 2023).

In summary, the salt affected to the growth of both *A. griffini* and *N. fowleri* trophozoites decreasing their multiplication rate as the salt concentration increased. Moreover, in a nutritive environment, the temperature also had an impact on the culture, confirming that at higher temperatures such as 28 and 37°C the growth of trophozoites increased. These results may contribute to the development of new control techniques of the amoebic populations in recreational water facilities.

## Data availability statement

The original contributions presented in the study are included in the article/Supplementary material, further inquiries can be directed to the corresponding authors.

## Author contributions

IA-J: Writing – original draft, Writing – review & editing, Data curation, Investigation, Methodology, Software. RR-E: Data curation, Investigation, Methodology, Software, Writing – original draft, Writing – review & editing. IS: Data curation, Investigation, Methodology, Software, Writing – original draft, Writing – review & editing. JC-P: Methodology, Software, Writing – original draft, Writing – review & editing. LS: Funding acquisition, Writing – original draft, Writing – review & editing, Formal analysis, Validation. AU: Formal analysis, Funding acquisition, Validation, Writing – original draft, Writing – review & editing. JP: Conceptualization, Funding acquisition, Project administration, Resources, Supervision, Validation, Visualization, Writing – original draft, Writing – review & editing. JL-M: Conceptualization, Funding acquisition, Project administration, Resources, Supervision, Validation, Visualization, Writing – original draft, Writing – review & editing.
